# Training Simulators for Gastrointestinal Endoscopy: Current and Future Perspectives

**DOI:** 10.3390/cancers13061427

**Published:** 2021-03-20

**Authors:** Martina Finocchiaro, Pablo Cortegoso Valdivia, Albert Hernansanz, Nicola Marino, Denise Amram, Alicia Casals, Arianna Menciassi, Wojciech Marlicz, Gastone Ciuti, Anastasios Koulaouzidis

**Affiliations:** 1 The BioRobotics Institute, Scuola Superiore Sant’Anna, 56025 Pisa, Italy; arianna.menciassi@santannapisa.it (A.M.); gastone.ciuti@santannapisa.it (G.C.); 2 Center of Research in Biomedical Engineering, Universitat Politècnica de Catalunya, 08034 Barcelona, Spain; albert.hernansanz@upc.edu (A.H.); alicia.casals@upc.edu (A.C.); 3 Department of Excellence in Robotics & AI, Scuola Superiore Sant’Anna, 56127 Pisa, Italy; 4 Gastroenterology and Endoscopy Unit, University Hospital of Parma, University of Parma, 43126 Parma, Italy; pcortegosovaldivia@ao.pr.it; 5 Department of Medical and Surgical Sciences University of Foggia, 71121 Foggia, Italy; nicola_marino.544176@unifg.it; 6 LIDER-Lab, DIRPOLIS Institute, Scuola Superiore Sant’Anna, 56025 Pisa, Italy; denise.amram@sns.it; 7 Department of Gastroenterology, Pomeranian Medical University, 71-252 Szczecin, Poland; wojciech.marlicz@pum.edu.pl; 8 The Centre for Digestive Diseases Endoklinika, 70-535 Szczecin, Poland; 9 Department of Social Medicine & Public Health, Pomeranian Medical University, 71-252 Szczecin, Poland; akoulaouzidis@hotmail.com

**Keywords:** GI endoscopy, medical simulation, medical education, training, gastroscopy, colonoscopy, simulators

## Abstract

**Simple Summary:**

Over the last decades, visual endoscopy has become a gold standard for the detection and treatment of gastrointestinal cancers. However, mastering endoscopic procedures is complex and requires long hours of practice. In this context, simulation-based training represents a valuable opportunity for acquiring technical and cognitive skills, suiting the different trainees’ learning pace and limiting the risks for the patients. In this regard, the present contribution aims to present a critical and comprehensive review of the current technology for gastrointestinal (GI) endoscopy training, including both commercial products and platforms at a research stage. Not limited to it, the recent revolution played by the technological advancements in the fields of robotics, artificial intelligence, virtual/augmented reality, and computational tools on simulation-based learning is documented and discussed. Finally, considerations on the future trend of this application field are drawn, highlighting the impact of the most recent pandemic and the current demographic trends.

**Abstract:**

Gastrointestinal (GI) endoscopy is the gold standard in the detection and treatment of early and advanced GI cancers. However, conventional endoscopic techniques are technically demanding and require visual-spatial skills and significant hands-on experience. GI endoscopy simulators represent a valid solution to allow doctors to practice in a pre-clinical scenario. From the first endoscopy mannequin, developed in 1969, several simulation platforms have been developed, ranging from purely mechanical systems to more complex mechatronic devices and animal-based models. Considering the recent advancement of technologies (e.g., artificial intelligence, augmented reality, robotics), simulation platforms can now reach high levels of realism, representing a valid and smart alternative to standard trainee/mentor learning programs. This is particularly true nowadays, when the current demographic trend and the most recent pandemic demand, more than ever, the ability to cope with many patients. This review offers a broad view of the technology available for GI endoscopy training, including platforms currently in the market and the relevant advancements in this research and application field. Additionally, new training needs and new emerging technologies are discussed to understand where medical education is heading.

## 1. Introduction

Medical simulators are artificial platforms that offer the opportunity to train clinical procedures in a non-patient care environment. The first endoscopic simulator is dated back to 1969 and was made up of a simple mannequin for sigmoidoscopy training [1]. Over the following fifty years, the technology advancements led to the development of several artificial platforms, targeting the training of a wide range of endoscopic procedures, including both the upper and the lower gastrointestinal (GI) tracts. Ranging from purely mechanical systems to more complex mechatronic devices and animal-based models, nowadays, a variety of opportunities are available for practicing cognitive and motor skills in endoscopy. For this reason, the American Society for Gastrointestinal Endoscopy (ASGE) has encouraged the use of simulators for GI endoscopy training [2].

Mastering endoluminal procedures requires a high level of visuomotor coordination, cognitive learning, and technical capabilities acquired through hands-on experience. The standard training system includes the mentoring of the trainee by an expert endoscopist. In this context, the novice progressively learns to manage a procedure by first assisting the expert clinician and later directly practicing on the patient [3]. However, this system implies several drawbacks. First, since endoscopy usually involves only one operator at a time, the trainee practices the procedure independently, even if under the supervision of mentors. This could increase the risks of causing discomfort or injuries on the patient, given the lack of experience of the operator [4]. Studies have shown an increased frequency of minor adverse events related to trainee procedures associated with patient’s dissatisfaction [5]. Moreover, in this context, if the novice encounters difficulties, the expert takes over the procedure, depriving the trainee of learning the specific task. Finally, this learning model leads to an increase of the length of the medical operation, negatively impacting on the costs and potentially prolonging the discomfort of the patient [4].

By contrast, simulators provide a risk-free solution for acquiring global competencies in medical practices at the trainee’s pace. Accordingly, as opposed to standard mentored supervision, simulation-based training allows trainees to repeatedly perform a specific set of skills without increasing the length of the clinical procedures and/or reducing the patient’s comfort and safety. Besides, these platforms could allow the standardization of the endoscopy related metrics and the assessment of technical skills [4,6]. This is particularly important for endoscopy since the outcome of the procedure is highly dependent on the skills and the experience of the operator [7,8]. Hence, the introduction of simulators in training programs would lead to evidence of the acquisition of the minimum competencies by the clinicians before operating on the patients [3,9]. In this context, the simulators would not only assess the minimum skills for trainees but could also serve to prove the maintenance of those skills to always guarantee high-quality patient care.

Over the years, various studies have estimated the minimum number of procedures needed to achieve competence in GI endoscopy. These values range from 100–300 for colonoscopy [10,11] and around 200 for gastroscopy [12]. In this context, simulation-based training could reduce this number, speeding up the learning curve of the trainee [9].

Currently, there are several commercial platforms available for GI endoscopy training. The present review classifies them based on the main characteristics into three categories: (i) mechanical simulators (Section 3.1), (ii) computerized simulators (Section 3.2), and (iii) animal models, both in-vivo and ex-vivo (Section 3.3). The platforms covered by this paper target either or both the upper and the lower GI tract, including procedures such as endoscopic retrograde cholangiopancreatography (ERCP), endoscopic ultrasonography (EUS), endoscopic mucosal resection (EMR), and endoscopic submucosal dissection (ESD). Moreover, non-commercial platforms are also presented (Section 4) in order to provide the reader with insights about the progress made by the research community in this field. Accordingly, the final goal is to present a broad view of the technologies available for training in GI endoscopy. To this end, the review paper focuses on highlighting both the beneficial aspects of the devices and the critical ones. In this context, the role of artificial intelligence (AI) and robotics is also discussed as an advanced solution to address the unmet challenges.

## 2. Fundamentals of Training in GI Endoscopy

**Figure 1:**
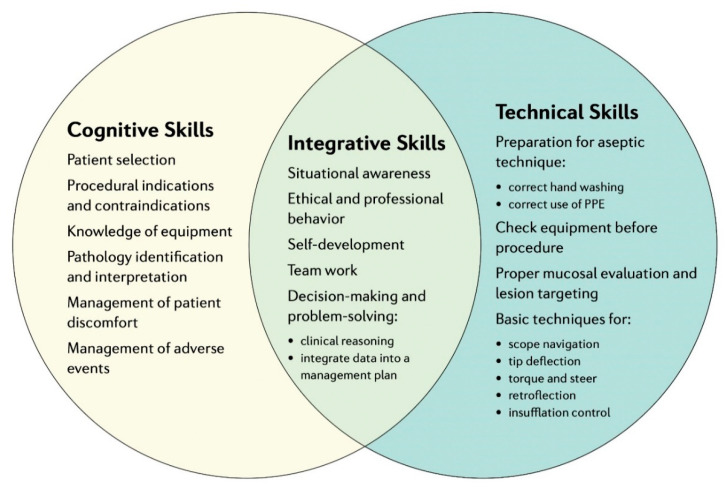
Skills needed for acquiring competency in gastrointestinal (GI) endoscopy—adapted from Walsh et. al. [15]. PPE: Personal Protective Equipment.

Defining endoscopic competence is a matter of continuous review, update, and scrutiny. High-quality care in gastrointestinal endoscopy is based not only on specific technical skills but also on cognitive skills and attitude, requiring, therefore, rather complex training and assessment. The importance of both technical and cognitive skills is highlighted by professional societies and regulatory bodies [13,14]. Required competencies that need to be developed and thoroughly assessed during training include technical and cognitive skills. The first include scope handling, strategies for loop reduction and advancement, and withdrawal and mucosal inspection, whereas the latter include clinical indication(s) for the procedure, clinical assessment, adverse event management, and knowledge of sedation protocols (Figure 1) [15,16].

The way to reach independent practice and expertise in GI endoscopy is mastered through continuous training, set in a framework that ideally includes progress monitoring, focused feedback, motivation enhancement, and instructional planning [17]. Nevertheless, assessment outcomes are not easy to determine and, thus, several tools, scales, and scores have been developed with this purpose, covering various domains of competence [15].

Great strides have been made with the introduction of simulators for training with a striking impact on the learning curve of novice endoscopists [18]. Nevertheless, many simulation-based endoscopy curricula still lack effective proficiency validation, as a standardized approach is missing. One step forward has been taken by Azzam et al. [16] who recently developed a comprehensive curriculum for the fundamentals of GI endoscopy (FGE). After choosing a model for outcomes (including both cognitive and technical skills), proficiency benchmarks were set, and novice endoscopists underwent a training module with the Accutouch simulator (see Section 3.2) with a level of proficiency pre-set at two standard deviations below the mean value of included experts. The outcome of the aforementioned study was that, at the end of the training, there was no statistical difference between the novice and the experts in pre-set outcomes (including scope navigation, landmark identification, biopsy forceps usage) regarding the ability to perform a safe procedure.

## 3. Conventional/Standard Simulators for Training

**Figure 2:**
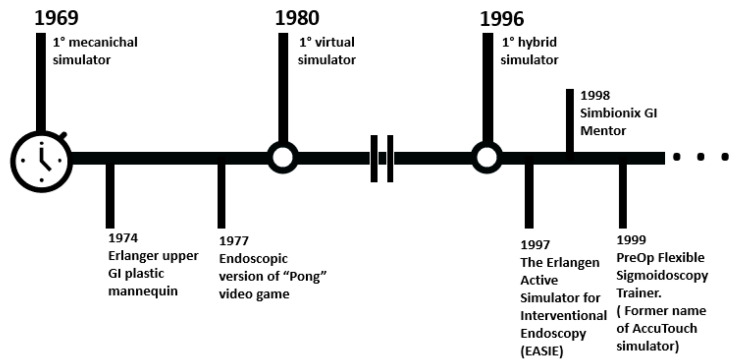
First steps of GI endoscopy simulators.

Over the years, several types of simulators for GI endoscopy training have been developed. Starting from the tests on animal models, technological progress enabled the development of the current generation of advanced devices (Figure 2). In particular, the training platforms for GI endoscopy can be classified, according to the technology adopted, into (i) physical simulators, (ii) computerized simulators, and (iii) animal models. The last category can be further differentiated into in-vivo and ex-vivo cases.

### 3.1. Mechanical Simulators for GI Endoscopy

Mechanical simulators (i.e., physical simulators), reproduce anatomical organs using a combination of soft and hard materials (e.g., silicone). In such cases, cavities inside the replicated phantoms allow the insertion of a standard endoscope, mimicking the endoscopic procedure. Consequently, the physical simulators aim at reproducing, with high fidelity, mechanical and visual properties of the GI tract, focusing on an accurate selection of the appropriate materials, molds, and surface textures. Even with the intrinsic advantage of providing natural tactile feedback, unfortunately, each platform offers only a limited set of training procedures, since each scenario needs to be physically reproduced. For this reason, the mechanical simulators are only suitable for early-stage training of GI endoscopy [19].

**Figure 3:**
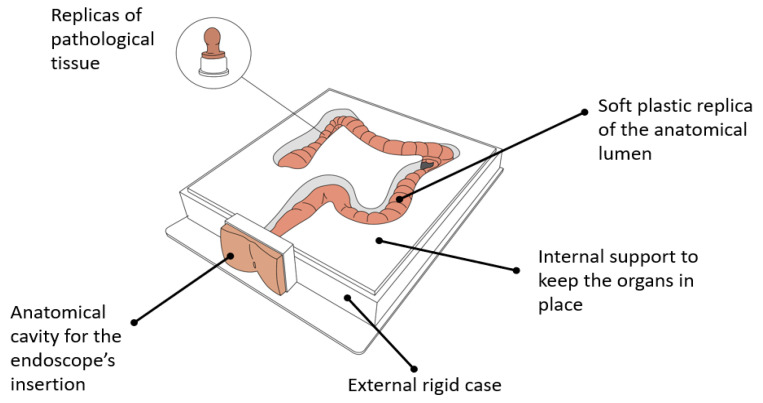
Mechanical simulators for GI endoscopy: main components.

Physical endoscopy trainers have been developed before the computerized platforms. One of the first works in this field was published in 1974, reporting the design of the Erlanger plastic mannequin, a training simulator for the upper GI tract [20]. Over the years, mechanical trainers have not experienced drastic changes. The main components of the current physical GI trainers currently on the market barely differ from those available in the Erlanger platform. These modules are listed below (Figure 3):

1. a replica of the anatomical lumen (i.e., esophagus, stomach, small intestine, colon) resembling the living organ in terms of visual appearance and tactile texture, made with soft plastic (e.g., silicone rubbers);
2. an external rigid case containing the phantom, endowed with one or multiple cavities, allowing the insertion of the endoscope (i.e., replicas of mouth, anal sphincter, nose);
3. rigid or semi-flexible internal support for keeping the organs in place, in some cases allowing the partial deformation/movement of the lumen during the procedure;
4. optional adds-on replicas of pathologic tissue (e.g., polyps) to be attached to the main organ, allowing one to practice multiple tasks (e.g., clipping, stenting, biopsy, polypectomy).

While virtual trainers simulate multiple procedures with the same platform (e.g., upper and lower GI endoscopy), physical trainers provide each module separately. The only comprehensive platform reproducing both colonoscopy and gastroscopy is the EMS Trainer (Chamberlain Group LLC, Great Barrington, Mass) [21]. However, this system allows one to reproduce only one scenario, has rigid support which does not leave space for large deformation of the organs, and replicates limited portions of the GI tract. Furthermore, just like all the other physical simulators, it does not provide any online suggestion, procedure’s guidelines, or objective measurements of the performance at the end of the training (Table 1).

**Figure 4:**
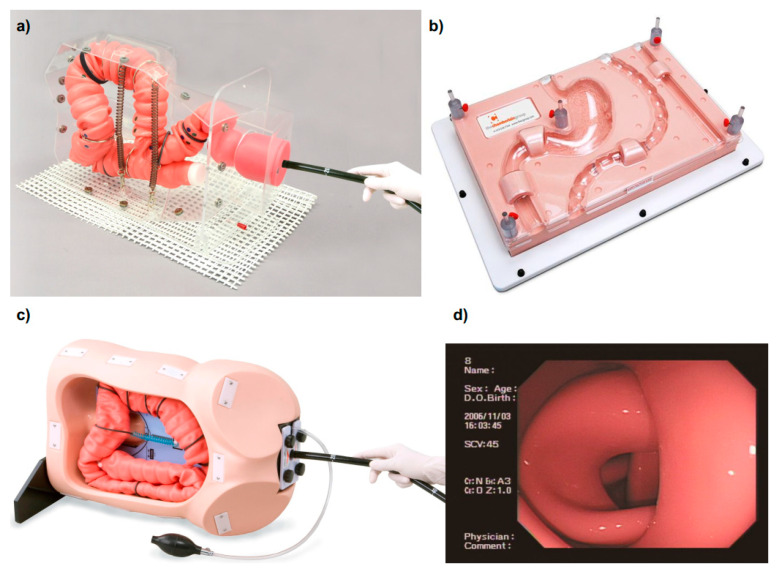
(**a**) Mechanical simulators: (a) MW24 NKS Colonoscope Training Simulator from Kyoto Kagaku Co. (Image supplied by Kyoto Kagaku Co.); (**b**) EMS trainer from Chamberlain Group, LLC (Image supplied by Chamberlain Group, LLC, copyright 2021); (**c**) M40 Colonoscope Training Simulator from Kyoto Kagaku Co. (Image supplied by Kyoto Kagaku Co.); (**d**) Internal endoscopic view of M40 Colonoscope Training Simulator from Kyoto Kagaku Co. (Image supplied by Kyoto Kagaku Co.).

Currently, three main medical companies produce mechanical GI trainers: the Chamberlain Group (previously mentioned), the Koken Co., Ltd. (Tokyo, Japan) [22], and the Kyoto Kagaku Co., Ltd. (Kyoto, Japan) [23] (Table 2, Figure 4). Besides the EMS Trainer [21], the Chamberlain Group produces also the Upper GI Trainer [24], the Biliary Endoscopy Trainer [25], and four types of Colonoscopy Trainer [26,27,28,29]. None of these platforms provides insufflation or suction, allowing the insertion of the scope through patient cavities. One of the colon trainers (i.e., Colonoscopy Trainer) replicates the shape of the colon and allows inserting a stricture and eight polyps in pre-selected locations [26], whereas the other two platforms (i.e., Colon Endoscopy Trainer with Flat Polyps and Colon Endoscopy Trainer with Raised Polyps) include a straight colon section, each featuring 25 polyps, respectively flat or raised, permanently embedded behind the replicated folds [28,29].

A more realistic environment for learning how to navigate the endoscope is provided by the two colonoscopy trainers of Kyoto Kagaku Co., Ltd. The first one, the Colonoscope Training Simulator [30], has the capability to make the colon tube air-tight and manipulate the anal sphincter opening using a hand air-pump. This system enables air-insufflation and suction. Different cases can be reproduced by easily modifying the configuration of the intestine and changing the colon fixtures, offering multiple levels of difficulties. The design of the colon tube and the support layout allows training for loops formation avoidance or straightening. Finally, applying a skin cover over the organs, manual compression may be practiced together with changing the position of the body (i.e., lateral or supine). The second platform produced by Kyoto Kagaku Co., Ltd., the 3D Colonoscope Training Simulator NKS [31], is even, i.e., more realistic than the first one, since it offers a three-dimensional representation of the colon based on an analytical study of computed tomography colonography. This simulator, particularly suitable to acquire navigation skills, including loop formation avoidance, allows one to pre-set the sigmoid colon to three different morphologies. The most suitable platform for training in polyp detection and removal are the Colonoscopy Lower GI endoscopy simulator type II [32] and the EGD Simulator [33], both produced by Koken Co., Ltd.. As a matter of fact, they allow to attach different types of polyps on the surface of the phantom including laterally spreading tumors in the ascending colon, gastric ulcers and early gastric cancer in the stomach and duodenum. In addition, polypectomy and clipping techniques can be trained, by attaching simulated tumors which bleeds once removed. 

Finally, it is worth mentioning that the Colonoscopy Lower GI endoscopy simulator type II by Koken Co., Ltd. is the only mechanical platform allowing one to train double balloon/single balloon enteroscopy [32]. This can be performed by attaching an additional replica of the small intestine to the main colonoscopy unit.

### 3.2. Computerized Simulators for GI Endoscopy

Considering what is accessible on the market, and besides mechanical simulators, another class of GI endoscopy training platforms is worth discussing. These devices, classified as computerized simulators (i.e., virtual reality simulators), are mechatronic systems that combine standard endoscope handling with virtual intraluminal scenarios. With that, it is possible to simulate a wide variety of endoscopic procedures and interventions (e.g., gastroscopy, colonoscopy, polypectomy, bleeding control ). Thanks to these platforms, the movements of a physical endoscope are mapped in a virtual environment, reproducing the endoluminal view with endoscopic images [18].

The first computerized simulators were developed in the 1980s as an adaptation of video games, reproducing EGD, colonoscopy, and ERCP [34,35]. At that time, the high cost of the technology did not facilitate their expansion into the clinical practice, which gradually arrived years later with the appearance of the GI Mentor (3D Systems, Littleton, Colorado, US) [36] and the CAE EndoVR Simulator (CAE Healthcare, Montreal, Quebec, Canada), previously called Accutouch [37].

**Figure 5:**
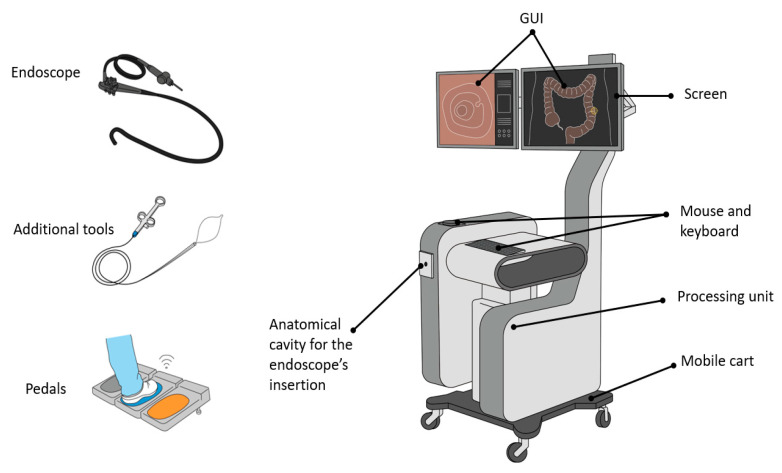
Computerized simulators for GI endoscopy: main components.

Nowadays, virtual reality (VR) simulators for intraluminal procedures are characterized by a combination of hardware components and software functionalities, aiming at training endoscopy beginners with the most realistic scenarios possible. Accordingly, the main physical modules included in these platforms are (Figure 5):

1. a mobile cart platform including one or two screens, a keyboard or/and a touchpad, a box with one or two anatomical plates (i.e., holes) for inserting the endoscope, and a processing unit;
2. a set of scope heads and tubes for upper and lower GI tract endoscopy with identical appearance and functionalities of those used in the clinical practice;
3. a collection of tools to insert in the endoscope operative channels (e.g., forceps, electrodes for coagulation); and
4. optional pedals for extra functionalities.

Concerning the software components, the VR simulators provide:

1. a graphical user interface (GUI) showing the simulated endoscopic environment together with all the additional information and aids regarding the procedure;
2. biomechanical simulation of the organs, allowing one to reproduce the expansion and the collapse of the lumen under insufflation or, in the case of a colonoscopy, looping formation;
3. haptic feedback mimicking the tactile sensation normally felt by the endoscopists while navigating the endoscope throughout a lumen;
4. a repository of real patient cases simulating diverse pathologies and anatomies with different level of difficulty both for upper and lower GI tracts;
5. indications for performance metrics both real-time and as a summary at the end of each procedure;
6. didactic modules providing online aids to the user such as step by step instructions on how to perform the procedure or a 3D map of the scope inside the lumen.

Having a wide variety of simulated cases in addition to the opportunity of reproducing the same scenarios for all the trainees is a great advantage for standardizing any type of training. All the platforms provide objective measurements of the quality of the performance such as the patient’s pain level, the percentage of mucosa visualized, the time of the examination, and the amount of air insufflated. This information allows tracking the learning curve of the users as well as customizing the benchmarks for the assessment of the capabilities of individual trainees. However, to have an immersive experience, visual and tactile rendering should be as realistic as possible. Otherwise, transferring skills from the simulator to real life could be a difficult task. In this regard, the realism of the tactile cues is closely related to the quality of the organ’s biomechanical model. A good approximation of the tissues’ behavior enables realistic force feedback computation.

**Figure 6:**
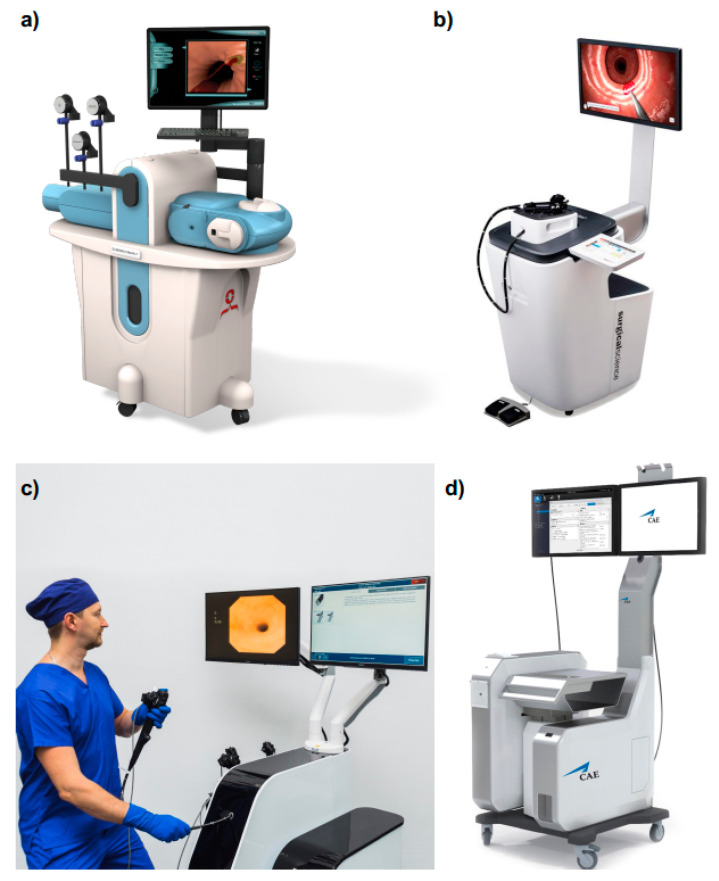
Computerized simulators: (**a**) Simbionix GI Mentor from 3D Systems (Image supplied by 3D Systems); (**b**) EndoSim from Surgical Science (Image supplied by Surgical Science); (**c**) EndoVison system from MedVision (Image supplied by MSE group); (**d**) CAE EndoVR from CAE Healthcare (Image supplied by CAE Healthcare).

Currently, in addition to the two aforementioned platforms (i.e., GI Mentor [36] and CAE EndoVR Simulator [37]), there are two more simulators commercially available for VR GI endoscopy training: (i) Endosim (Surgical Science, Gothenburg, Sweden) [38] and (ii) Endo Vision STANDARD (MedVision, Nihonbashi Honcho, Chuo-ku, Tokyo, Japan) [39] (Table 2, Figure 6). In addition, a third platform called Endo-X (Medical-X, Rotterdam, Netherlands) was commercialized until 2017, but it is now out of production [40,41]. However, the Endo-X features are reported in Table 2. Finally, Olympus (Olympus Keymed, Essex, UK) also developed a simulator for GI endoscopy training, i.e., Endo TS-1, but it was never commercialized [42].

Each of these platforms integrates the basic hardware and software components previously listed in this section with few variations on the training modules (Table 2). Indeed, all the VR simulators include both upper and lower GI endoscopy. However, the GI Mentor is the only platform including a module for EUS and one for ESD/EMD [36]. Moreover, it provides a couple of training tasks in a non-anatomical environment to learn the basics of endoscope manipulation. Finally, it also comprises a didactic set of training for guiding the user in the learning of the deconstructed skills as defined by the Society of American Gastrointestinal and Endoscopic Surgeons (SAGES), i.e., endoscopic navigation, mucosal evaluation, targeting, retroflexion, and loop reduction [13,36]. This application is performed with augmented reality (AR) cues on simulated intraluminal scenarios and by providing step by step instructions. Similar modules are offered by Endo-X [40] and Endosim simulators [38]. The latter also allows recording the user’s performance to be able to rewind them as a learning tool [38].

An additional interesting feature is offered by CAE EndoVR, which provides online indications of the patient vitals (i.e., heart rate, oxygen saturation, blood pressure) during a procedure simulation and asks the trainee to manage the sedation. Doing so provides a more immersive experience to the user [37].

Finally, a distinctive feature of the GI Mentor worthy to report is the possibility to have the simulator in a portable format called GI Mentor™Express [43]. This simulator includes a box for inserting the endoscope, and it can be plugged into every laptop, which is used as a screen.

### 3.3. Animal Models

Animal models have been widely exploited for training in medical procedures. For endoscopic applications, the literature dates the first ERCP simulation on a live canine model in 1974 [44]. Overall, both live animals and explanted organs have been used for practicing in GI endoscopic procedures. However, high costs and ethical concerns related to the in-vivo cases and the logistic issues in collecting and storing ex-vivo specimens limit their use. As a matter of fact, animals are mainly adopted for advanced training in therapeutic procedures and for reproducing complex techniques such as hemostasis [45,46], EUS [47,48,49,50], ERCP [51,52], and ESD [53,54,55,56,57].

In addition, limited evidence is available on the literature regarding the use of cadavers for training in GI endoscopy. This practice has been mainly dedicated to the training of advanced surgical techniques, e.g., ESD [58], per oral endoscopic myotomy (POEM) [59,60,61], or natural orifice transluminal endoscopic surgery (NOTES) [62]. The main advantage reported is the quality of the tactile feedback [58] that overcomes the one of the artificial simulation platform. However, cadaveric tissues present a different stiffness to live tissues, sometimes making the procedure harder to perform [61].

#### 3.3.1. In-Vivo Animal Models

Endoscopic training in in-vivo models consists of practicing the whole procedure or part of it on anesthetized live animals. Specific clinical scenarios, such as targeted lesions, can be artificially reproduced before starting the training [48]. According to the literature, large animals are the most used for training in endoscopy, since their GI tract dimensions most resemble the human ones. Doing so, the replicated procedure is performed with standard endoscopic devices. Among the animals, swine are the most commonly adopted for endoscopy training both in upper and lower GI tracts, in particular for EUS [47,48,49,50,51], ESD [53,54,55], and ERCP [51,63]. However, regarding the ERCP, diversities in porcine and human anatomy make biliary cannulation more difficult in pigs [52]. The same issue arises while attempting this procedure on dogs, making the canine model inadequate as well [44]. In this context, the most realistic approximation is represented by the baboon [64], even though their use for training purposes raises several ethical questions due to the similarities between humans and primates [65].

As reported, the adoption of live animal models for the training has been limited to the most complex procedures. As a matter of fact, on one hand, in-vivo cases provide real haptic feedback very close to the one experienced with human tissues as well as high visual rendering. Besides, they allow the trainer to practice drug administration, replicating all the conditions of the real clinical intervention: secretions, respiratory movements, and bleeding. With this regard, in case of tissue damage or bleeding, it is possible to check the tissue recovery eventually in a second endoscopic procedure. On the other hand, there are evident anatomical differences between humans and animals, reducing the realism of this kind of model and sometimes making it inadequate for the training. In addition, the need of on-site care facilities and veterinary staff for performing the procedure significantly increases the whole process’ costs. Finally, ethical concerns regarding the sacrifice of animals for clinical training purposes conservatively limit their use [31]. Indeed, endoscopic training as part of animal experimentation requires the careful approval of the planned protocol by specific independent institutions ensuring animal welfare as well as the compliance with applicable regulations and standards [66].

Nowadays, to pursue a risk-based approach that identifies roles and responsibilities, standards of skills and training for the dedicated staff and mechanisms of monitoring become essential preparatory activities to develop endoscopic training under the in vivo animal model.

In this regard, the World Organization for Animal Health (OIE) [67] refers to three key elements to enable the use of animals for research and education purposes: (i) the existence of a project/training proposal review developed under a risk-based approach, (ii) the identification of transparent inspections procedures of the facilities to ensure their suitability for the project/training, and (iii) the ethical evaluation of the overall procedures involving animals (e.g., methodologies, source of animals, staff’s skills and competence, husbandry, transportation) [68].

The mentioned OIE key elements are generally required in the documentation, and procedures have to be submitted to the competent independent body/committee/authority in several jurisdictions, even though different approaches might address specific obligations/limits in a given legal system. Therefore, the conformity of the use of animals for our educational purposes shall be assessed case-by-case.

For example, in the European Union, the Directive 2010/63/EU [69] established a series of principles and procedures that the Member States had to regulate in the national implementations. In particular, it presents a structured methodological approach to be replicated in the national legislative initiatives to enable a compliant—and, therefore, ethically accepted—use of animals in education and training. To this end, specific guidelines have been drafted by the Experts Working Group (EWG) appointed by the EU Commission to properly address the interpretations of the Directive [70].

In particular, the mentioned EU legal framework requires the researcher to provide a full exploration of alternative strategies through the identification of objectives and defined benefits within any request for the use of live animals [69]. In our scenario, indeed, this would include a list of alternative learning methods, e.g., virtual simulators, physical trainers, or ex-vivo models, and the reasons why they are inadequate for reaching the desired training goals.

National implementations may, however, concern possible limits and procedures to assess the justification required for the use of animals, in general, identified by applying the 3Rs criteria (replacement, reduction, and refinement) [71]. They shall also refer to specific skills and competencies for those who are designing and carrying out procedures involving animals. They shall include periods of supervision as well as specific mechanisms of continuing assessment of acquired skills and competencies. The acquisition of knowledge is not sufficient to proceed with in-vivo trials for education purposes [69,70]. This is particularly true in our field of application, where technological progress may always improve standards of accuracy and decrease the level of pain/invasive perception.

In addition, according to article 24 of the mentioned Directive, at least one person in the training center shall be appointed to take care of the animals and oversee their welfare [69]. These activities shall include daily checks on animals and the development of strategies to increase awareness of the culture of animal care within the whole staff. The appointed staff member must also act as a liaison between the training center and institutional bodies and other professionals (e.g., the Animal Care Body, the Veterinary Services, and experts able to recognize any variation on normal health and behavior) [70].

Moreover, according to the EWG report, the request for approval of the use of live animals for education and training shall address a series of issues [70], as illustrated in Table 3.

In light of the illustrated remarks, the in vivo model shall be developed in specialized and certified structures, firstly identifying the applicable ethical legal framework, then developing the procedures by addressing the binding requirements in light of the main benefits/risks assessment in each step of the procedures. The workflow and the proactive approach required to recall the one developed to deal with any other ethical issues that emerge in research activities, where principles other than the animals’ care and wellbeing are paramount to conduct the given research [70].

#### 3.3.2. Ex-Vivo Animal Models

Ex-vivo animal models for GI endoscopic training are composites systems, including a plastic frame and an explanted specimen. In this case, the desired lumen is inserted in a rigid case, which gives support to the soft tissues, mimicking the abdominal surrounding organs. The simulated procedure is performed by using standard endoscopic devices. These models are much easier to set up concerning live animals and represent a slight improvement from an ethical standpoint. Indeed, the GI samples are supposed to come from the slaughter industry, where the animals are killed for the meat industry.

The first simulator of this kind was developed in 1997 using porcine intestine’s specimens. Initially, it was called The Erlangen Active Simulator for Interventional Endoscopy (i.e., the EASIE model), while later it started to be commercially distributed as the Erlanger Endo-Trainer model (ECE-Training GmbH, Erlangen, Germany) [72,73,74]. The system includes a plastic structure reproducing a human head and torso, in which the explanted upper GI samples can be installed. Depending on the procedure to be simulated, the specimen undergoes different specific preparation (e.g., the recreation of polyps, small lesions, band ligation, tumors, varices, strictures). The rigid case, replicating the torso, can be rotated around the longitudinal axis and can be fixed in any lateral position. Realistic bleeding is rendered with a perfusion device endowed with an adjustable container and a stop-valve system. The blood circulation is regulated by an electric pump simulating the heart rate of the patient, and it is easily controlled by an assistant.

A more compact version of the Erlanger Endo-Trainer was later developed, called the Erlangen compact EASIE/EASIE-R (EndoSim, LLC, Bolton, Mass) [46,75,76]. In this case, all the hardware parts are reduced to a small rigid frame for the fixation of the organ and a roller pump for the hemostasis simulation. Despite the reduced size, it allows one to perform up to 30 different procedures. Nowadays, there are different variations of the compact EASIE commercially available. The most recent one, EASIE-R4, adapted for the upper GI tract, includes a torso-shaped tray with attachment clamps to fix the specimen in position. This device is endowed with support for the esophagus, the stomach, and a portion of the duodenum. An extra insert for the biliary ducts is also included for the training of ERCP and EUS. The ERCP module enables the simulation of fluoroscopy, avoiding the use of X-rays. Furthermore, a specific frame called the COLOEASIE-2, suitable only for colonoscopy replicas, has been designed. In all these platforms, the biological specimens can be supplied by local butchers or by specialized companies with particular competencies in harvesting and preparing the tissues. In this case, the animal organs may be frozen and delivered to be ready for installation into the models after thawing.

As with the compact EASIE, two other types of composite simulators are available on the market: the Endo X Trainer (Medical Innovations International, Rochester, Minn) [77] and the DeLegge EndoExpert Tray (DeLegge Medical LLC, Awendaw, SC) [78] for training in the both upper and lower GI tracts.

Overall, ex-vivo animal models have the advantage to provide more realistic haptic and visual feedback compared with mechanical and virtual simulators. However, using explanted organs, the tissue characteristics may change compared to the live ones (e.g., loss of elasticity), increasing the difficulty of endoscope navigation. As an advantage, the costs are moderate, especially in comparison with computerized simulators. Nevertheless, the tissues require long preparation and appropriate disposal, and the numbers of training scenarios are intrinsically limited. Finally, as with mechanical simulators, no online guidelines or quality final metrics are provided during the training.

## 4. GI Endoscopy Simulators in Research

In addition to commercial platforms, there is a consistent number of training simulators at a research level. This set of devices includes mechanical, computerized, and hybrid systems. A part of them presents similar characteristics to those available on the market, whereas others show distinctive features. In particular, several developed platforms aim at reducing the costs and the dimensions of the device to ease their widespread distribution and promote the uptake of simulation among the endoscopy units.

As a result, many researchers have focused on the development of mechanical simulators made with easy-to-find and inexpensive materials (e.g., Polyvinyl chloride (PVC) hose, derange tubes, plastic sheaths, latex balloons, plastic boxes). In these systems, the anatomical phantom is fixed to the desk mainly using rubber bands, and polyps or biopsy sites are replicated with small pieces of sponges, foam padding, or snap fasteners. The construction of these platforms is meant to be easy and achievable by many people without any technical background [79,80,81]. A more realistic but still cost-effective system was developed by exploiting the 3D printing technology for the manufacturing of the upper GI tract replica [82]. The organs’ 3D models are reconstructed from several computed tomography (CT) images of neck, chest, and gastrography using free software such as 3D Slicer (3D reconstruction) [83] and Autodesk Meshmixer (mesh modification) [84].

A more complex platform was recently developed by Fuji et al. based on a hybrid colonoscopy simulator. The system called “Mikoto” is an advanced mechanical trainer endowed with motors, which allow the position change of the replicated abdomen. Furthermore, several functions are supported, such as abdominal compression and repositioning of the diaphragm, simulating the deep inspiration. Pressure and optical sensors enable measuring the distensibility of the colon phantom correlated with the potential pain experienced by the patient. This information together with other metrics tracked online is used to evaluate the trainee performance [85].

Regarding virtual simulators, the research mainly focuses on improving the realism of the systems and creating low-cost portable platforms. Trade-offs need to be found between realistic force feedback, organ and endoscope deformation, visual rendering, and computational cost [86,87,88,89,90,91]. A promising example of this kind of simulators is the one developed by the Australian Commonwealth Scientific and Industrial Research Organisation (CSIRO) for colonoscopy. In this system, the endoscope is inserted in a small box, which is easily connected to a laptop that is used as a monitor for the training. Realistic visual rendering and haptic feedback improve the quality of the training, still keeping the device’s dimensions modest [92].

Lastly, another set of simulators that have been investigated focus on the training of tip control in non-realistic environments. In this case, the design does not aim at reproducing in detail the procedure but at creating adequate conditions for improving the trainer’s endoscope control. Accordingly, the simulator developed by Riek et. al. includes only a series of interchangeable hemispherical training surfaces containing a sequence of circular “targets” and a clamp designed to hold a standard colonoscope. The endoscope tip is maintained at a fixed distance from the concave training surface without impeding tip flexion and torque. The system is endowed with software that visually assists the training and recognizes when a target is reached [93].

A similar platform was developed for training the tool’s tip positioning in ERCP. The mechanical system, called Boškoski-Costamagna ERCP Trainer, consists of a metal framework with plastic anatomical organs, and various papillae made with latex. The trainer, which does not provide realistic visual feedback, allows one to practice different ERCP procedures (i.e., scope insertion, wheel and elevator handling, selectively cannulating bile and pancreatic duct, stone extraction, and both metal and plastic stent insertion) [94].

## 5. Validation of GI Simulators

An important aspect related to the introduction of any simulation platform in training programs or more generally in the market is their validation. In this regard, several studies that can be conducted to provide evidence of the performance of the simulators at different levels. The first stage of assessment is the “face validity”, a type of study usually performed at the earliest phases of the devices’ development. In this context, a team of experts is asked to score the performances of the simulator in terms of ability to teach and evaluate what it intends to teach and assess. Following this, the “content validity” is a second subjective assessment in which experts evaluate the realism of the simulation with respect to the live one. In this case, each component of the simulator is analyzed in detail and scored. Thirdly, the “construct validity” is performed to assess the ability of the device to discriminate the levels of expertise of the operators. Finally, the highest level of evidence is provided by the “predictive or transfer validity”, aiming at proving the ability to transfer the skills learned on the trainer to the real practice on patients [95,96].

In this regard, in 2012, ASGE published an initiative called “Preservation and Incorporation of Valuable endoscopic Innovations (PIVI)”, providing two metrics as a validation tool for GI endoscopic trainers [2]. To this end, ASGE suggests that the introduction of a specific simulator into a training program is justified if its use by novices leads to a reduction of 25% of the clinical cases needed to learn the procedure. Accordingly, several studies have been conducted on the different trainers aiming at proving the advantages of simulation-based training as compared with the standard one. A comprehensive list of the relevant works in this field for upper and lower GI tracts are reported in [97].

The second metric introduced in the PIVI as a validation tool is related to the ability of the simulators to assess the skills of the user. The simulation platform can also be used for procedural credentialing. In particular, it was decided that the simulator’s assessment should correlate with the operator’s competences in live endoscopy with a kappa value of at least 0.70 [2,96]. In this case, although several studies have tested the construct validity, few have computed the requested correlation [97,98,99,100].

## 6. Experts’ Commentary and Lesson Learned

### 6.1. Clinically Oriented

One of the key factors in assessing the success of simulators is their actual implementation within resident training and continuing education curricula in the medical units of hospitals with training programs. Especially now, with the new reality of the pandemic setting in, early-stage training and retaining/enhancing the basic skills in an endoscopist’s curriculum is particularly pertinent. However, several aspects interfere with wider implementation. The first one is the discussion about clinical validity and the long-term usefulness of training with simulators. This argument is not so notable with physical platforms but becomes germane with virtual devices due to their interactive training interface and overall skills assessment capabilities; “simulator behavior” is not the same with the real-life endoscopy room performance, as important elements of the interaction are missing. Soft skills such as standard phrases and instructions are not in focus, and staff resource management cannot be practiced and checked. Moreover, there is a lack of objective studies on any improvements obtained by using relevant training platforms within well-structured curricula.

Another aspect that slows down the implementation of virtual simulation platforms in replacing classical patient-based training methodologies is the economic trade-off between the acquisition of such platforms (generally expensive) versus the economic expense per healthcare system. Nevertheless, the relationship between experience and operating theater throughput (shorter surgeries) as well as patient morbidity rate (safer surgeries) is clear. The costs associated with operating theater usage have also been studied [101]. Furthermore, as the current dynamics of in-patient training have proven to be functional, the implantation of simulators is difficult. The benefits obtained are not direct and are therefore difficult to quantify, resulting in a barrier to entry. [102,103]. The cost of a computerized simulator ranges from $50,00 up to $135,00 depending on the number of modules purchased. Additional costs for the maintenance of the system must be considered as well [97]. However, simulators could also be rented instead of purchasing them, eventually reducing the associated costs.

Overall, more objective studies are still required to evaluate and quantify the cost/benefit ratio of established simulators in terms of (i) operating theater productivity [104], (ii) probability of reintervention and morbidity, and (iii) their associated costs. Different medical societies that are committed to generating standardized training and evaluation courses as well as the generation of guidelines for correct training.

### 6.2. Technologically Oriented

Besides the aforementioned issues, a contributing factor to the limited deployment of virtual platforms is their lack of visual and physics realism rendering. Both represent a barrier against the user immersion during training sessions and a gap between training and real scenarios. The physics simulation of tissue deformation, tool interaction, and suture thread is still an open field.

It is still not uncommon to observe physical breakages of rendered volumes due to excessive deformation, unrealistic dynamic behavior due to the difficulty of modeling and calculating the behavior of the tissues in real-time, unrealistic gravity simulation in suturing threads, etc. The continuous advances in parallel computing in graphical computer units (GPUs) with an affordable price are contributing to decrease this problem. The evolution of open-source physics computation libraries and packages is also contributing to increasing the simulation realism. Examples of these software packages to compute and render physics are SOFA (Simulation Open Framework Architecture) [105], NVIDIA PhysX [106], Bullet [107], etc. In addition, high-level graphical packages such as Ogre [108], Unity [109], etc., have opened the possibility to develop realistic visual rendering.

On the other hand, mechanical simulators and ex-vivo models can easily provide more realistic tactile and visual feedback. However, they are unable to reproduce multiple procedures with the same platform, and they provide little information about quality metrics, e.g., trainee evaluation or learning curve. To this end, limited examples are available in the literature reporting cases of sensorized physical platforms used to collect measures (e.g., contact forces) during the procedure [110,111].

A similar consideration can be done for in-vivo animal models, which have the potential of realistically replicating the clinical scenarios, but their use brings many ethical and economical concerns. To this end, nowadays, training on live animals is limited only to very complex procedures, e.g., ESD, in the case that no other simulation can be used instead. However, the continuous technological progress will strongly decrease, if not eliminate, the need to use animal models for educational purposes, e.g., it may be achieved by enhancing accurate virtual reproduction of scenarios and tasks (see Table 3).

Therefore, the authors conclude that the next generation simulators need to focus on pure virtual environments, avoiding the use of explanted tissues and live animal models for ethical issues but also guaranteeing quantitative metrics for immediate evaluation and continuous monitoring and comparison of performance (i.e., to precisely calculate the learning curve). This is possible today thanks to the improvement in computer vision, artificial intelligence, and augmented/virtual reality techniques and stressing biomechanical modeling at local and macro levels to properly mimic properties of organs fed back to the user through multi-sensing haptic feedback. However, for extensive adoption of simulators in hospitals, clinical staffs need to accept and endorse their use within training programs, consequently breaking any barriers left to embrace technologically driven education solutions.

## 7. Conclusions and Future Perspectives

Learning is a lifelong process, and “perfection” is achieved by continuous practice. Simple and fundamental measures, e.g., the number of colonoscopies required to reach competency—defined by a cecal intubation rate (CIR) ≥90%—are often not well established and are not enough to evaluate learning [112,113]. Assumptions, generalizations, and approximations which are remnants of the past remain in practice to date, and quantitative measures are needed. However, there is a change upon us, and there are conditions that will facilitate this. First, there is the popularization of medico-legal litigations that has, to an extent, enforced not only accountability but also the need to reinforce delivery strategies; second, there is the emergence and the galloping of AI for improving the realism and the efficacy of the clinicians training in gastroenterology and medicine overall that allows for training options and possibilities that were not there at the start of the millennium; third, there is the looming singularity—humanity’s last frontier, i.e., the time when the abilities of a computer overtake the abilities of the human brain that does not allow compliance; last but not least, events such as the most recent pandemic highlight the weaknesses of the healthcare systems in not only its inability to cope with large numbers of patients but also to provide constant and uninterrupted training to the next generations of clinicians, particularly gastroenterologists/endoscopists. Turbulent waves of gastroenterology-related complications are expected due to the diversion of healthcare resources at the time of COVID-19 [113]. Although the data on the impact of the current pandemic on endoscopy training are scarce, several international surveys aimed to address this issue have recently been reported [114]. In general, the COVID-19 pandemic has resulted in a reduction of the number of endoscopies in many countries followed by restricted endoscopy training in in-vivo conditions and higher rates of anxiety and burnout among trainees [114,115,116]. These restrictions on medical specialty training programs are predicted to persist at least for the next several years [117]. Not surprisingly, national GI societies (e.g., the British Society of Gastroenterology) have already been engaged in seeking solutions. Examples of such initiatives include the proposals for optimization of learning curves for cognitive skills by facilitating access to hands-off methods and simulation-based training [11,116,117]. With this in mind, we must move beyond standard learning processes and engage in risk-free solutions utilizing medical simulators at various levels of clinical practice. This way of high-level education could be maintained, which will guarantee the high quality of patient care.

In addition to the wide description of training models, classifications, and the current work carried out in the educational scientific field, it is mandatory to understand where medical education is heading following new training needs and new emerging technologies.

Demographic trends such as the climb in the global population, the increase of life expectancy, and the population density in favor of the elderly drive the needs for care and access to health services and surgical procedures [118]. At the same time, the advancement of social and individual well-being, especially in the most populous countries such as China and India [119], has induced profound cultural and lifestyle changes, paving the way for a process of westernization that today affects the prevalence of diseases [120], primarily neoplastic and cardiovascular, typical of countries with high well-being.

The above will have a profound impact on requests for access to highly specialized medical procedures [121], hence the need to train an ever-increasing number of operators. This will require optimization of learning curves by adopting standardized training protocols developed in collaboration with international scientific societies and new models of practical training to be adopted as early as possible.

On the other hand, the exponential advancement of technologies, thanks to the growth trend of the computational capacity of microprocessors and therefore the reduction of volume and cost for the same performance according to Moore’s law, allows one today to imagine new applications and respond to new needs like never before [122].

The new training tools will have to respond to specific requirements; not only will they have to follow strict development methodologies [123], but they will also need to integrate digital technologies and be affordable, feasible, and available—in one word, democratic.

Any training system must therefore consider the need to acquire data and analyze them with algorithms [124] to monitor performance improvement, motivation, and planning of the learner’s teaching while overcoming the “physical” limits of academies and exporting a shared knowledge within a global community. Monitoring the learning curve will also be essential in case of medico-legal disputes [125], allowing one to objectify the acquisition of basic specific skills together with the update of practical knowledge.

It is therefore assumed that the future of practical education will aim to adopt hybrid models capable of mixing increasingly realistic environments with new digital tools, Internet of Things devices, and artificial intelligence algorithms.

The next goal is to democratize access to education and automating and objectifying the acquisition of practical skills, thus reducing the impact of costs derived from physical tutors and expensive training centers. This model could be defined as “phygital” as the fusion of physical and digital qualities properties and is being promoted by a few companies, such as INTECH (intechsimulation.com, Milan, Italy), that believe in the possibility to spread medical education by lowering costs with the support of technology.

Moreover, the practical training of the near future will have to integrate an analysis of non-technical skills [126], such as the ability of the operator to respond promptly and with clarity to the emergence of adverse events unexpected and capable of endangering the patient’s health. This topic overcomes the classic considerations regarding learning curves in the sense of acquiring surgical skills, as it includes emotional aspects that might interfere with the regular practice and eventually alter its outcomes. It is therefore easy to think that, just as in aviation or space fields, also in medical training will the use of neural interfaces [127] be used for the measurement of stress, fatigue, and performance of healthcare workers to receive biofeedback and personalize training in real-time to gain and monitor results that include an emotional point of view and ultimately to increase patient safety.

Finally, the authors envisage in the future the introduction of new robotic platforms to perform gastroscopy and colonoscopy [111,128,129]. Therefore, following parallelism with the introduction of the daVinci robot as an alternative to conventional minimally invasive surgical procedures, new simulators may be designed and introduced for training on future robotic-assisted GI endoscopy, even if there is not a gold standard today. Or, if the combination of AI and robotics in the development of new medical platforms will make the clinical procedure easy and, in some cases, automatized or semi-automatized, training simulators may not be necessary in the future. This is still an open question, and more questions follow in conclusion: (1) are we going to expect new advanced training simulators in the future as a consequence of the improvement of AI- and AR/VR-based techniques or of the introduction of new robotic platforms? or (2) are we going to foresee a sunset of them in the next few years due to the better compliance or the new robotic platforms for GI endoscopy?

## Figures and Tables

**Table 1:** Advantages and disadvantages of each simulator.

Type of Simulators	Advantages	Disadvantages
**Mechanical**	Low-cost Realistic force feedback	Limited set of training procedures per platform Lack of educational feedback or online suggestions Lack of quality final metric and tracking of the users’ improvements Useful only for early-stage training
**Computerized**	Multiple simulated procedures, anatomies and patients’ cases available in the same platform Possibility of reproducing the same scenario for all the trainers Delivery of educational feedback and online suggestions Measurement of the performances and tracking of users improvements∙ Inclusion of drugs management	Non-realistic force feedback Non-realistic visual rendering High cost
**Ex-Vivo Model**	Realistic force feedback High visual rendering Low cost	Limited set of training procedures per platform Lack of educational feedback or online suggestions Lack of quality final metric and tracking of the users’ improvements Difficulty in collecting and storing the specimens
**In-Vivo Model**	Realistic force feedback High visual rendering Realistic scenario Useful for training advanced procedure	Animal anatomy is different form human anatomy High costs Ethical concerns Need of on-site-care facilities and veterinary staff

**Table 2:** Comparison of modules and key-features of the different GI simulators.

Type of Platform	Virtual Simulators					Mechanical Simulators								Ex-vivo Simulators		
Simulators	GI Mentor	CAE Endo VR	Endo-X	EndoSim	EndoVision	Endoscopic Trainer for the upper GI system Chamberlain Group	Biliary Endoscopy Trainer Chamberlain Group	Colonoscopy Trainer Chamberlain Group	EMS trainer Chamberlain Group	Colonoscope Training Simulator Kyoto Kagaku Co.	3D Colonoscope Training Simulator NKS Kyoto Kagaku Co.	Colonoscopy Lower GI endoscopy simulator type II Koken Co.	EGD Simulator Koken Co.	EASIE-R4 EndoSim,	ColoEASIE-2 EndoSim,	Erlanger Endo-Trainer
**Modules**																
Upper GI endoscopy	**✓**	**✓**	**✓**	**✓**	**✓**	**✓**	**x**	**x**	**✓**	**x**	**x**	**x**	**✓**	**✓**	**x**	**✓**
Lower GI endoscopy	**✓**	**✓**	**✓**	**✓**	**✓**	**x**	**x**	**✓**	**✓**	**✓**	**✓**	**✓**	**x**	**x**	**✓**	**✓**
Non-anatomical environments	**✓**	**x**	**x**	**x**	**✓**	**x**	**x**	**x**	**x**	**x**	**x**	**x**	**x**	**x**	**x**	**x**
ERCP	**✓**	**✓**	**x**	**✓**	**x**	**x**	**✓**	**x**	**x**	**x**	**x**	**x**	**✓**	**✓**	**x**	**✓**
Flexible sigmoidoscopy	**✓**	**✓**	**✓**	**x**	**x**	**x**	**x**	**x**	**x**	**x**	**x**	**x**	**x**	**x**	**✓**	**✓**
EUS	**✓**	**x**	**x**	**x**	**x**	**x**	**x**	**x**	**x**	**x**	**x**	**x**	**x**	**✓**	**x**	**✓**
GI Bleeding	**✓**	**✓**	**✓**	**x**	**✓**	**x**	**x**	**x**	**x**	**x**	**x**	**✓**	**✓**	**✓**	**✓**	**✓**
EMR/ESD	**✓**	**x**	**x**	**x**	**x**	**x**	**x**	**x**	**x**	**x**	**x**	**x**	**x**	**x**	**✓**	**✓**
Polypectomy/Biopsy	**✓**	**✓**	**✓**	**✓**	**✓**	**x**	**x**	**x**	**x**	**x**	**x**	**✓**	**✓**	**✓**	**✓**	**✓**
Bronchoscopy	**✓**	**✓**	**x**	**✓**	**✓**	**x**	**x**	**x**	**x**	**x**	**x**	**x**	**x**	**x**	**x**	**✓**
Enteroscopy	**x**	**x**	**x**	**x**	**x**	**x**	**x**	**x**	**x**	**x**	**x**	**✓**	**x**	**x**	**x**	**✓**
Basic skills	**✓**	**✓**	**✓**	**✓**	**✓**	**✓**	**✓**	**✓**	**✓**	**✓**	**✓**	**✓**	**✓**	**✓**	**✓**	**✓**
**Key features**																
Force feedback	**✓**	**✓**	**✓**	**✓**	**✓**	**✓**	**✓**	**✓**	**✓**	**✓**	**✓**	**✓**	**✓**	**✓**	**✓**	**✓**
Intestinal looping	**✓**	**x**	**x**	**✓**	**x**	**x**	**x**	**x**	**x**	**✓**	**✓**	**x**	**x**	**x**	**x**	**x**
Multiple organs layout	**✓**	**✓**	**✓**	**✓**	**✓**	**x**	**x**	**x**	**x**	**✓**	**✓**	**x**	**x**	**✓**	**x**	**✓**
Replicas of polyps	**✓**	**✓**	**✓**	**✓**	**✓**	**x**	**x**	**✓**	**✓**	**x**	**x**	**✓**	**✓**	**✓**	**✓**	**✓**
Replicas of ulcers	**✓**	**✓**	**✓**	**✓**	**✓**	**x**	**x**	**x**	**✓**	**x**	**x**	**x**	**✓**	**✓**	**✓**	**✓**
Replicas of strictures	**✓**	**✓**	**✓**	**✓**	**✓**	**x**	**✓**	**✓**	**✓**	**x**	**x**	**x**	**x**	**x**	**✓**	**✓**
Suction/insufflation	**✓**	**✓**	**✓**	**✓**	**✓**	**x**	**x**	**x**	**x**	**✓**	**✓**	**x**	**x**	**✓**	**✓**	**✓**
Virtual patients’ cases	**✓**	**✓**	**x**	**x**	**✓**	**x**	**x**	**x**	**x**	**x**	**x**	**x**	**x**	**x**	**x**	**x**
Patient vitals measurements	**✓**	**x**	**x**	**x**	**x**	**x**	**x**	**x**	**x**	**x**	**x**	**x**	**x**	**x**	**x**	**x**
Multiple body positions	**✓**	**✓**	**✓**	**✓**	**✓**	**x**	**x**	**x**	**x**	**✓**	**✓**	**x**	**x**	**x**	**x**	**✓**
Manual abdominal compression	**n/a**	**n/a**	**n/a**	**n/a**	**n/a**	**x**	**x**	**x**	**x**	**✓**	**✓**	**x**	**x**	**x**	**x**	**x**
Drugs management	**✓**	**✓**	**✓**	**x**	**x**	**x**	**x**	**x**	**x**	**x**	**x**	**x**	**x**	**x**	**x**	**x**
Online tips	**✓**	**✓**	**x**	**x**	**✓**	**x**	**x**	**x**	**x**	**x**	**x**	**x**	**x**	**x**	**x**	**x**
3D Map of the organ	**✓**	**x**	**x**	**✓**	**✓**	**x**	**x**	**x**	**x**	**x**	**x**	**x**	**x**	**x**	**x**	**x**
Recording the procedure	**✓**	**x**	**x**	**✓**	**✓**	**x**	**x**	**x**	**x**	**x**	**x**	**x**	**x**	**x**	**x**	**x**
Trainee feedback	**✓**	**✓**	**✓**	**✓**	**✓**	**x**	**x**	**x**	**x**	**x**	**x**	**x**	**x**	**x**	**x**	**x**

n/a: not applicable; ERCP: Endoscopic Retrograde Cholangio-Pancreatography; EUS: Endoscopic Ultrasonography; EMR: Endoscopic Mucosal Resection; ESD: Endoscopic Submucosal Dissection, EGD: Esophagastroduodenoscopy.

**Table 3:** This table illustrates how to justify the GI endoscopic training scenario under the requirements for caring for animals aiming for better science [70].

Issue to be Addressed	Possible Remarks in GI Endoscopic Training
**Learning objectives**	Hands-on practice of the most complex procedures in GI endoscopy, e.g., ESD, in the case no other simulation environments can correctly replicate the procedure.
**Target group for the training activity**	Advanced trainees (not beginners)
**Purposes and needs of the procedures**	Learn a specific endoscopic procedure, which cannot be practiced elsewhere (i.e., using other types of simulators) and that is too dangerous to perform on patients without previous experience.
**The procedure is a single demonstration/trainee will directly participate**	Trainees directly practice the procedure.
**Justification of the in-vivo model respect to alternative models, by exploring other teaching methods highlighting the reasons of their unsuitability (e.g., comparing with experiments on human volunteers, video- and computer-based learning methods, and in vitro and ex-vivo studies)**	Cases in which a realistic haptic feedback very close to the one experienced with human tissues and high visual rendering are paramount for correctly training the procedure. Therefore, even the most advanced simulators and ex-vivo models cannot replicate a scenario close enough to the real one.
	When it is extremely important to practice, together with the technical procedure, drug administration, replicating all the conditions of the real clinical intervention: secretions, respiratory movements, and bleeding. However, advanced virtual and physical simulators now allow repetitive practice of the clinical tasks in highly realistic environments.
**Specify whether the procedure could be video recorded to avoid replicating the use of animals**	Watching recorded procedures helps in learning the technique, although hands-on practice is preferred and usually paramount for acquiring technical skills.
**Prior methodologies and approaches before applying in-vivo work**	Watching recorded or live procedures performed by expert physicians. Test specific set of technical skills on simulators or ex-vivo models.
**Reasons that avoid the learning objective to be fulfilled by observation of on-going research**	Technical skills are hard to learn by only watching experts performing the specific tasks. Therefore, trainees usually need to acquire them directly on the field.
**Justify the procedure in terms of severity if greater than mild**	Before starting to simulate an endoscopic procedure, the animals are anesthetized and sacrificed at the end of the training. Although this protocol does not induce severe pain on the animals, refinement techniques should be always applied to minimize the suffering.
**What feedback will be sought from the students on whether the educational objectives have been attained**	Students will be able to perform the procedure on patients with a previous successful experience on animal models. However, similar experience can be gained by repetitive training the complex tasks on different types of simulators or ex-vivo models.

ESD: endoscopic submucosal dissection.

## Abbreviations

GI	Gastrointestinal
ASGE	American Society for Gastrointestinal Endoscopy
ERCP	Endoscopic Retrograde Cholangio-Pancreatography
EGD	Esophagastroduodenoscopy
EUS	Endoscopic Ultrasonography
EMR	Endoscopic Mucosal Resection
ESD	Endoscopic Submucosal Dissection
AI	Artificial Intelligence
FGE	Fundamentals of GI Endoscopy
VR	Virtual Reality
AR	Augmented Reality
SAGES	Society of American Gastrointestinal and Endoscopic Surgeons
PIVI	Preservation and Incorporation of Valuable endoscopic Innovations
POEM	Per Oral Endoscopic Myotomy
NOTES	Natural Orifice Transluminal Endoscopic Surgery
